# Explaining the colour of natural healthy gingiva

**DOI:** 10.1007/s10266-024-00906-4

**Published:** 2024-02-25

**Authors:** Cristina Gómez-Polo, Javier Montero, Ana Maria Martín Casado

**Affiliations:** 1 Department of Surgery, School of Medicine, University of Salamanca, Campus Miguel de Unamuno s/n, 37007 Salamanca, Spain; 2 Department of Statistics, School of Medicine, University of Salamanca, Salamanca, Spain

**Keywords:** Spectrophotometer, Natural gingival colour, Caucasian population, Regression models, Gender

## Abstract

To examine the differences between natural gingival colour in men and women. To determine the degree of predictability of changes in the gingival colour coordinates recorded for healthy gingiva, according to age, long-term medication, frequency of toothbrushing, and smoking habits. The CIELAB colour coordinates were recorded using a spectrophotometer for 360 Caucasian adult participants (aged 18–92 years), in three zones of the healthy attached gingiva of the maxillary central incisor. Regression models were created for each zone and each sex, taking the L*, a* and b* coordinates as dependent variables and age, frequency of toothbrushing, smoking habits (0—non-smoker; 1—smoker) and whether participants were taking long-term medication (0—no; 1—yes) as independent variables. The statistical analysis was conducted with SPSS version 26.0, using multiple regression models. Statistically significant differences between men and women were found only for colour coordinate b*, in all three zones. The only colour coordinate on which the predictor variables had a significant effect was the L* coordinate. In men, age and long-term medication had the greatest effect as predictors (maximum R^2^ = 0.149). In women, frequency of toothbrushing was the strongest predictor in the predictive models (maximum R^2^ = 0.099). The colour of gingiva in men contained a larger amount of blue, given that significantly lower values for colour coordinate b* were recorded in men than women, although this difference lacked clinical implications. For both sexes, the regression models produced had a modest predictive capacity. The L* coordinate was the dependent variable that showed the greatest predictability.

## Introduction

The attractiveness of a person’s face is linked closely to the beauty and harmony of the smile, which is one of the most important expressions for transmitting emotions and feelings [1, 2]. Even at a social level, an attractive smile is positively related to variables connected to personality, success in interpersonal relations, higher professional status, greater academic performance, and better future employment prospects [3].

An appealing smile requires a well-balanced facial composition, taking into account the hard and soft facial tissues (particularly the lips), configuration of the teeth (position, size, shape and colour) and their relation to gingival tissues [4]. Other authors [5, 6] have identified that altered gingival colour negatively affects people’s assessments of attractiveness, as well as kindness, decency, and intelligence. The importance of dental and gingival aesthetics therefore goes beyond beauty, affecting perceptions in the social world. That is why such emphasis is placed on creating functional, well-integrated prosthetic restorations which closely resemble natural teeth: to help individuals project a positive image and ensure that restorations provide a satisfactory result, particularly in the anterior region.

Achieving gingival aesthetics for prosthetic restorations that are as close as possible to the surrounding gingiva is complicated by a number of factors. These include the wide inter-individual and intra-individual variability of gingival dimensions (width, height, thickness, texture) [7, 8], the fact that gingival replacement techniques and materials have not developed as fast as those applied to the teeth [9, 10], the breadth of the gingival colour space [10–12], subjects’ view that colour is one of the most important factors in aesthetic perceptions [3, 13, 14], and the fact that gingival colour changes have still not been quantified.

It should be underlined that natural gingival colour is determined by multiple factors, which can be either exogenous or endogenous. It is influenced by race [15–20], the intensity of melanin pigments [21–23], sex [17, 24, 25], age [25], location [10, 26, 27], and gingival biotype [28], as well as the degree of vascularisation [21, 29–31], frequency of toothbrushing [10], smoking [11, 32–34] and whether the subject takes medication [35–37].

The use of verbal descriptions of gingival colour such as *pale pink*, *coral pink* or *dark pink* [11, 21] is not useful or reliable for communicating information about a colour or reproducing it. There are not enough studies providing evidence of the correspondence between these verbal descriptions of “pink” colours and the natural gingival colour. Similarly, manufacturers have failed to produce spectrophotometric results proving that the gingival colour range they offer corresponds to the actual range of natural gingival colours. In the absence of a “gold standard” gingival shade guide [10, 38], communication and reproduction of the gingival colour chart for the papilla and the three areas of attached gingiva is closely connected to the artistry of the dental technician in mixing basic gingival colours.

Studies predicting colour changes have now taken on particular importance [39], whether concerning in vitro evaluation of dental materials [40] or in vivo research on teeth [41], but there are no studies on the estimated colour range in gingival tissue. Gaining an objective understanding of how the colour of natural, healthy gingiva is influenced by sociodemographic and behavioural variables (frequency of toothbrushing, smoking habits, medication, etc.) is vital to enable customisation of the colour of prosthetic restorations, and can also help diagnose local and systemic diseases that have chromatic effects on the mucosa (e.g., gingival infections, chronic desquamative gingivitis, mucous membrane pemphigoid and gingival hyperplasia). It is significant that colour is one of the four signs by which inflammation has conventionally been identified (heat, pain, redness and swelling). While it has been suggested that certain exogenous factors can influence the gingival colour of men and women, none of the existing literature examines gender differences when exploring the differential relationships between certain variables and gingival colour coordinates.

It is worth noting that studies have been published to calculate dental colour coordinates according to sex and age, using regression models [41, 42], but there are no publications that address gingival colour. If we were able to estimate the colour changes that occur naturally in the gingiva, this information would help us more accurately select the gingival colour. Over time, it would therefore be possible to make any colour differences between natural gingival tissue and prosthetic gingival restorations as subtle as possible (clinically acceptable).

The objectives of this study are: (1) to study the differences between natural gingival colour in men and women; (2) to determine the degree of predictability of changes in the gingival colour coordinates recorded for healthy gingiva in an adult population, according to age, sex, long-term medication, frequency of toothbrushing, and smoking habits.

The null hypotheses of this study are: (1) that there are no statistically significant differences between the colour coordinates in the two sexes; (2) that the factors analysed have no explanatory power with regard to any of the colour coordinates of the gingiva in the three zones studied, in either sex.

## Material and methods

This study has been approved by the Bioethics Committee of the University (CBE.USAL.16/15). In the university’s dental clinic, voluntary subjects who met the following inclusion criteria were recruited: (1) presenting with two healthy maxillary teeth; (2) aged between 18 and 92 years; (3) not presenting with gingival pigmentation; (4) having healthy gingival tissue; (5) of Caucasian race; and (6) having signed the informed consent form.

A Spectroshade Micro (MHT Optic Research) spectrophotometer was used, which had first been tested for accuracy and reliability in recording the colour coordinates. Three colour readings were taken in each gingival zone analysed (the free gingival margin [FGM], middle zone and mucogingival line) for every participant, making a total of nine chromatic measurements. The arithmetic means of the colour coordinates provided by the spectrophotometer (L*, a* and b*) were then used in the statistical analysis. To quantify colour, the Commission Internationale de l’Éclairage (CIE, International Commission on Illumination) created a uniform colour space called CIELAB, on the basis of three colour coordinates, as follows. The L* coordinate corresponds to the vertical axis and refers to the lightness of the colour (from black at 0, to white at a value of 100); the a* coordinate, on a horizontal axis, refers to redness (for positive values of a*) or greenness (for negative values); while the b* coordinate, which is also on a horizontal axis, indicates how yellow (when positive) or blue (when negative) the colour is [43].

All the colour readings were taken under standardised conditions and after calibration of the spectrophotometer: each subject, who had removed all make-up, rested their head on the same dental cabinet, under fluorescent daylight-colour lamps (Philips TLD 58/965), without resting the spectrophotometer on the tissue, and without drying the area of analysis with the air syringe. All colour readings were taken by the same operator—aged 30, with eight years of clinical experience—who had received prior theoretical and practical training on gingival colour.

In order to satisfactorily assess the individual effects of each explanatory variable, controlling for the effects of the other variables, we then proceeded to fit the multiple regression models [44]. For each sex, the three regression models were fitted in each of the gingival zones, taking the L*, a* and b* coordinates as dependent variables, and age, frequency of toothbrushing, smoking habits (0—non-smoker; 1—smoker) and whether the subject had been taking medication for over 6 months (0—no; 1—yes) as independent variables. All of the statistical analyses were conducted using IBM’s SPSS software, version 26.0.

## Results

### Descriptive statistics

There were 360 participants in the study: 187 men (51.9%) and 173 women (48.1%), aged between 18 and 92 years (mean age 47.2 years; SD 18.8 years). No statistically significant difference was found between the ages of the men and women who participated in the study (t = 1.407, P = 0.160). With regard to the behavioural factors: 61 of the participants were smokers (16.9%), while 299 were not (83.1%); 102 were taking long-term medication (28.3%), and 258 were not (71.7%); and the mean frequency of toothbrushing was 2.0 times per day (SD 0.8) (Table 1).

**Table 1:** Number of participants for each category of the variables analysed

Variable	Frequency	%
Sex		
Male	187	51.9
Female	173	48.1
Age		
30 years or younger	89	24.7
From 31 to 45 years	85	23.6
From 46 to 60 years	89	24.7
From 61 to 75 years	72	20.0
75 years or older	25	6.9
Frequency of toothbrushing (per day)		
0	2	0.6
1	110	30.6
2	133	36.9
3	111	30.8
4	4	1.1
Smoking habits		
Smoker	61	16.9
Non-smoker	299	83.1
Long-term medication		
Yes	102	28.3
No	258	71.7

### Analytical statistics resulting from multivariate analyses of variance (MANOVA)

The study’s findings on the relationship between the aforementioned variables were as follows: (1) a statistically significant, negative correlation between age and the frequency of toothbrushing in the sample as a whole (r = − 0.450, P < 0.001) and in the subsamples of men (r = − 0.511, P < 0.001) and women (r = − 0.356, P < 0.001); (2) a statistically significant difference in the age of the participants who were taking long-term medication and those who were not (entire sample—61.9 vs 41.3, P < 0,001; subsample of men—62.0 vs 42.3, P < 0.001; subsample of women—61.7 vs 40.4, P < 0.001); (3) a statistically significant difference between the frequency of toothbrushing of the participants who were taking long-term medication and of those who were not (entire sample—1.6 vs 2.2, P < 0.001; subsample of men—1.6 vs 2.1; subsample of women—1.7 vs 2.2, P < 0.001); (4) a statistically significant difference between the frequency of toothbrushing in men and women (1.9 vs 2.1, P = 0.024).

Table 2 shows the mean and standard deviation for the colour coordinates in each zone of the gingiva, according to sex, as well as the P value for the multivariate analyses of variance (MANOVA) that were conducted to find out whether there were statistically significant differences between the colour coordinates for men and women.

**Table 2:** The mean (SD) values for L*, a* and b* by gingival zone and sex, and the P value for the comparison of colour coordinates in men and women

Attached gingiva	Men Mean (SD)			Women Mean (SD)			F; P value^a^
	L*	a*	b*	L*	a*	b*	
Mucogingival line	49.8 (6.4)	23.7 (4.0)	**14.5 (2.5)**	49.5 (5.9)	23.5 (4.3)	**15.2 (2.8)**	F = 2.771; 0.042
Middle zone	50.3 (5.8)	24.4 (4.1)	**14.7 (2.5)**	50.9 (5.6)	24.0 (4.1)	**15.8 (3.1)**	F = 5.354; 0.001
FGM	49.8 (6.2)	24.1 (4.3)	**14.5 (2.5)**	50.9 (5.6)	23.6 (4.5)	**15.3 (2.7)**	F = 3.833; 0.010

The coordinate in which differences were identified is highlighted in bold text

^a^The values that appear in this column are those obtained in the three one-way MANOVAs conducted in each zone of attached gingiva to compare the colour coordinates in men and women. When these were significant (in this case, in all three gingival zones), an analysis was conducted to identify which of the three coordinates differed significantly between men and women. The significant univariate effects are in bold

**Fig. 1.**
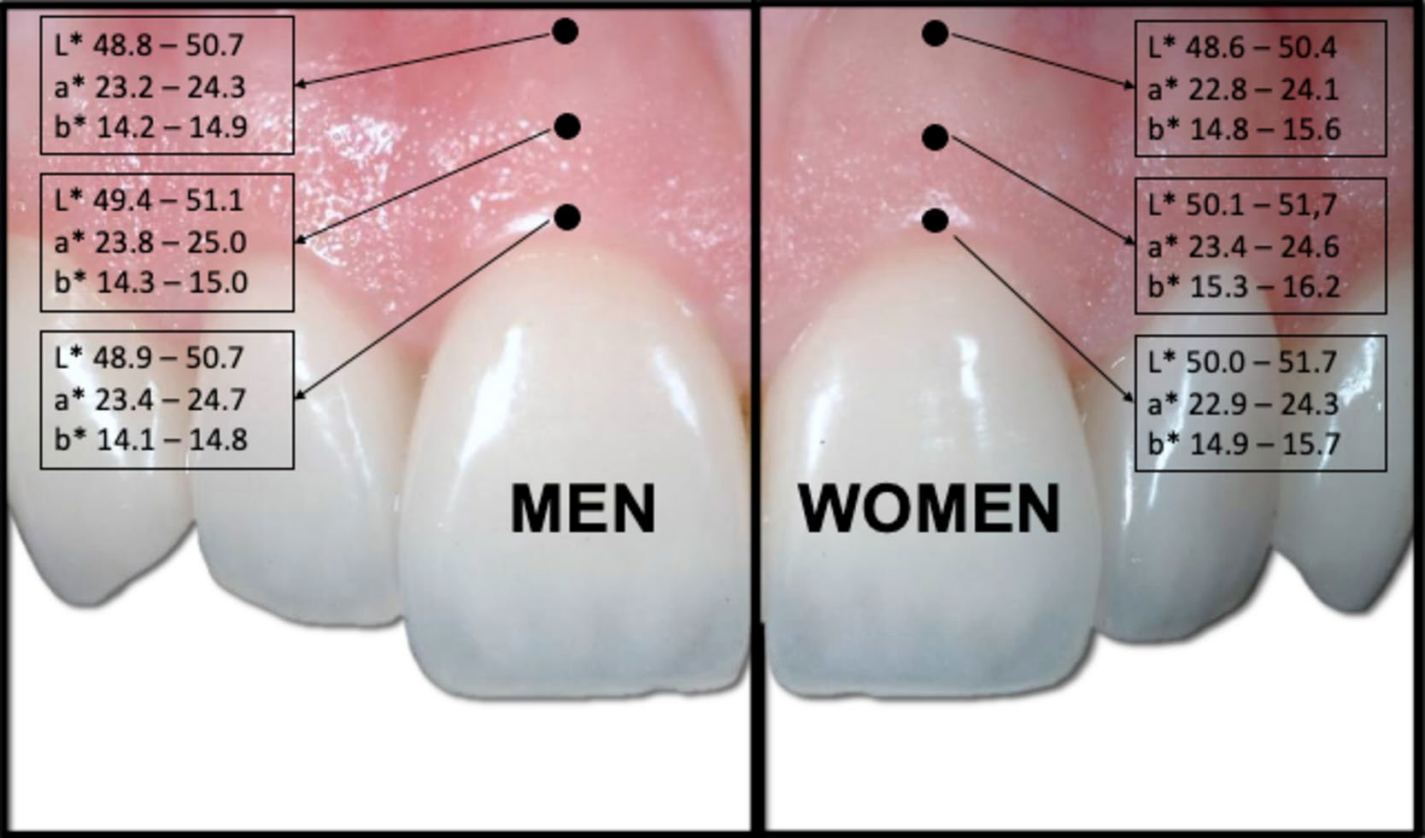
95% confidence interval for the L*, a* and b* coordinates in each gingival zone, according to the gender

As the table shows, there were statistically significant differences between men and women in the three gingival zones, all of which were due to variations in the b* coordinate (Fig. 1).

### Multiple linear regression analyses

**Fig. 2:**
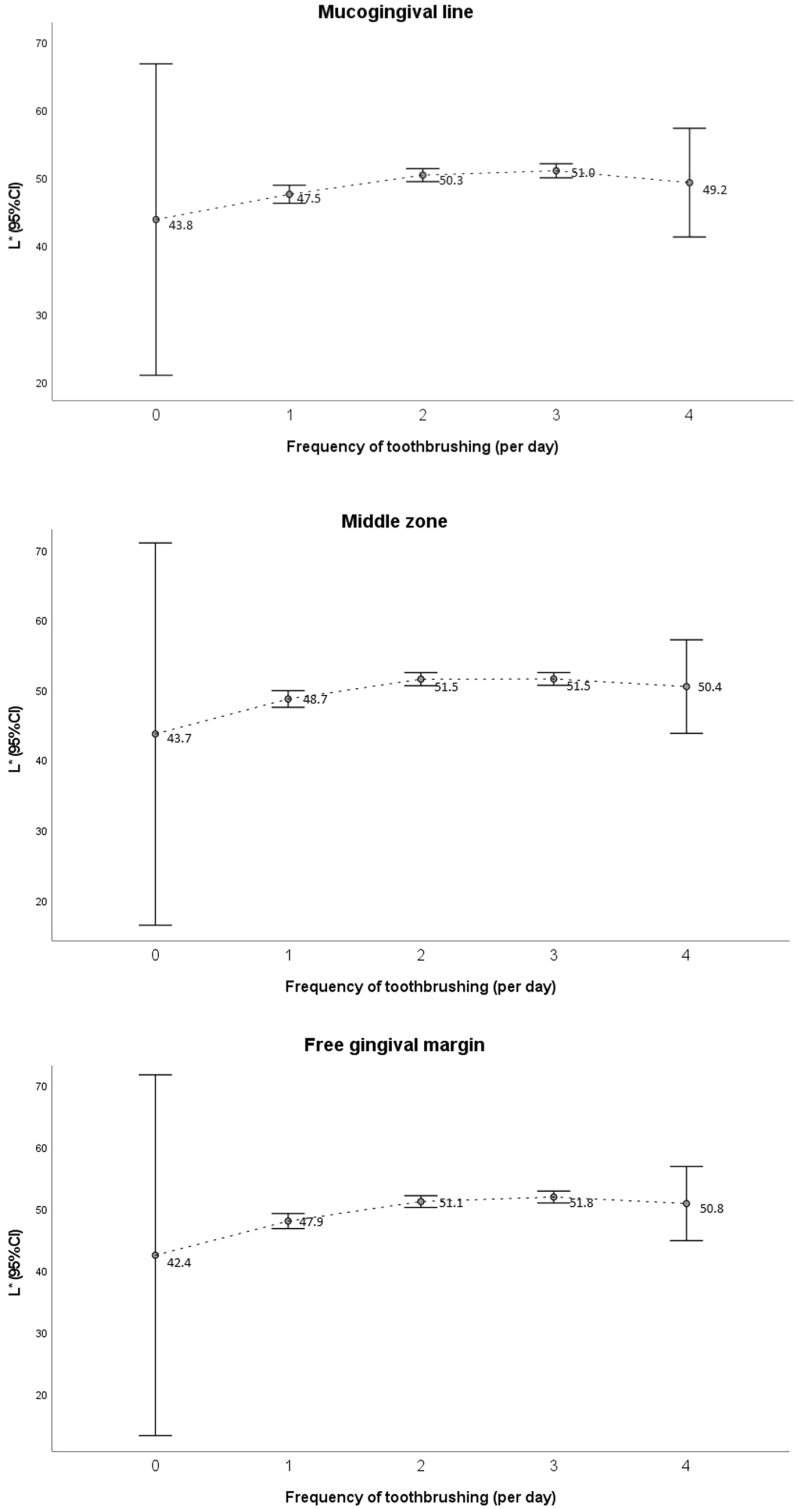
Mean and 95% confidence interval for the L* coordinate in each gingival zone, according to the frequency of toothbrushing

Regression models were fitted, taking age, frequency of toothbrushing, smoking habits and long-term medication as predictor variables. The fitted regression models had low coefficients of determination. In order to improve them, the square of the frequency of toothbrushing was incorporated into the model, since this predictor seemed to have a quadratic effect on the colour coordinates, as illustrated in Fig. 2 for coordinate L* [10].

Figure 2 shows that the effect of frequency of toothbrushing on the L* coordinate is not linear. For example, an increase from brushing 0 times per day to 1 time per day (which increases the mean value for L* by approximately 4 or 5 units, depending on the gingival zone) is not the same as an increase from brushing two to three times per day (which increases the mean for L* by approximately 0–1 unit).

The fitted multiple regression models for each sex are provided below.

#### Regression models for men

Table 3 shows the standardised regression coefficients of the fitted models for the subsample of men (n = 187) in each of the gingival zones, the P-value of the test for significance of regression, and the coefficients of determination.

**Table 3:** Standardised regression coefficients (in bold, the statistical significance of the regression model, P < 0.05) of the fitted models according to gingival zone, for men

	Mucogingival line			Middle zone			Free gingival margin		
	L*	a*	b*	L*	a*	b*	L*	a*	b*
Age	0.230	− 0.091	− 0.069	0.157	− 0.064	− 0.086	0.092	− 0.132	− 0.026
Frequency of toothbrushing (per day)	0.193	− 0.597	− 0.100	0.051	− 0.061	− 0.120	0.112	− 0.061	0.083
(Freq. toothbrushing)^2^	0.204	0.580	0.066	0.237	0.020	0.023	0.205	− 0.007	− 0.080
Smoking habits	0.028	− 0.038	0.090	0.004	0.034	0.098	− 0.056	0.030	0.056
Long-term medication	− 0.112	0.067	− 0.106	− 0.196	0.129	− 0.094	− 0.210	0.063	− 0.100
Sig. of the regression	**< 0.001**	0.764	0.388	**0.001**	0.684	0.318	**< 0.001**	0.786	0.684
R^2^	0.128	0.014	0.028	0.102	0.017	0.032	0.149	0.013	0.017

The only colour coordinate upon which the factors studied have a significant effect is the L* coordinate, with a medium effect size at the mucogingival line and in the middle zone and a large effect size at the free gingival margin [45]. The fitted regression models in men were:

Mucogingival line:

$${L}^{*}=42.165+0.074\cdot Age+1.444\cdot {\textit{Frequency of toothbrushing}} \left(per day\right)+0.377 \cdot {{\textit{Frequency of toothbrushing}}}^{2}+0.488\cdot Smoking habits-1.536\cdot {\textit{Long term medication}}$$

(range of prediction errors from − 16.2 to 15.3; residual standard deviation 6.0).

Middle zone:

$${L}^{*}=46.447+0.045\cdot {\textit{Age}} +0.346\cdot {\textit{Frequency of toothbrushing}} \left({\textit{per day}}\right)+0.394{\cdot {\textit{Frequency of toothbrushing}}}^{2}+0.069\cdot {\textit{Smoking habits}}-2.433\cdot {\textit{Long term medication}}$$

(range of prediction errors from − 14.8 to 14.0; residual standard deviation 5.5).

Free gingival margin:

$${L}^{*}=46.280+0.028\cdot {\textit{Age}}+0.804\cdot {\textit{Frequency of toothbrushing}} \left(per day\right)+0.364{\cdot {\textit{Frequency of toothbrushing}}}^{2}-0.944\cdot {\textit{Smoking habits}}-2.771\cdot {\textit{Long term medication}}$$

(range of prediction errors from − 14.8 to 14.9; residual standard deviation 5.7).

In men, age and the behavioural habits explain 12.8% of the variation in the L* coordinate at the mucogingival line of the attached gingiva, 10.2% of its variation in the middle zone and 14.9% at the free gingival margin [44].

At the mucogingival line, the factors that most affect the L* coordinate are age and the frequency of toothbrushing. For example, the expected increase in the L* coordinate when age increases by 10 years (holding the rest of the explanatory variables constant) is 0.7 (95% CI from 0.2 to 1.3). In other words, the gingiva becomes lighter with age in the upper zone. For frequency of toothbrushing, the expected increase in the L* coordinate when the frequency increases from 2 to 3 times per day (keeping the rest of the explanatory variables constant) is 3.3 (95% CI from 1.3 to 5.4). In both situations, the L* coordinate increases, meaning that the attached gingiva in the upper area becomes lighter. In the middle zone, the factors that most affect the L* coordinate are: the frequency of toothbrushing, taking long-term medication, and age. The expected increase in the L* coordinate when age increases by 10 years (keeping the rest of the explanatory variables constant) is 0.4 (95% CI from − 0.1 to 1.0), and when frequency of toothbrushing increases from 2 to 3 times per day (keeping the rest of the explanatory variables constant), it increases by 2.3 (95% CI from 0.5 to 4.2). In both cases, the middle part of the attached gingiva becomes lighter. At the free gingival margin, the factors that contribute most to explaining the variation in the L* coordinate are long-term medication and the frequency of toothbrushing (quadratic effect). The expected increase in the L* coordinate when participants take long-term medication (keeping the rest of the explanatory variables constant) is − 2.8 (95% CI from − 4.8 to − 0.8). This means that taking long-term medication results in a darkening of the free gingival margin. In contrast, the free gingival margin becomes lighter when the frequency of toothbrushing increases from 3 to 3 times per day (keeping the rest of the explanatory variables constant): the L* coordinate increases by 2.6 (95% CI from 0.7 to 4.6).

#### Regression models for women

Table 5 shows the standardised regression coefficients of the fitted models for the subsample of women (n = 173) in each of the gingival zones, the P value of the test for significance of regression (the statistically significant models with P < 0.05 are highlighted in red), and the coefficients of determination.

**Table 5:** Standardised regression coefficients (in bold, the statistical significance of the regression model, P < 0.05) of the fitted models according to gingival zone, for women

	Mucogingival line			Middle zone			Free gingival margin		
	L*	a*	b*	L*	a*	b*	L*	a*	b*
Age	− 0.042	− 0.101	− 0.140	0.008	− 0.109	− 0.161	− 0.027	− 0.095	− 0.051
Frequency of toothbrushing (per day)	1.043	− 1.151	0.970	1.217	− 0.967	0.858	0.981	− 0.586	0.995
(Freq. toothbrushing)^2^	− 0.957	0.963	− 1.040	− 1.112	0.808	− 0.881	− 0.868	0.418	− 0.982
Smoking habits	− 0.076	− 0.027	0.069	− 0.080	− 0.013	0.010	− 0.151	0.104	0.110
Long-term medication	0.056	− 0.038	0.015	0.062	− 0.018	0.047	0.024	− 0.063	0.002
Sig. of the regression	**0.020**	**0.015**	**0.020**	**0.005**	0.068	**0.050**	**0.003**	0.062	0.068
R^2^	0.076	0.081	0.077	0.096	0.059	0.064	0.099	0.061	0.059

In women, the only factor that had an important effect on the colour coordinates, in all three zones, was the frequency of toothbrushing. At the mucogingival line, in contrast to the results in men, this factor affected all three coordinates, not only L*, although the effects on all of them were small. In the middle zone, it had only a moderate, significant effect on the L* and b coordinates; and at the free gingival margin, it had only a moderate, significant effect on the L* coordinate [45].

The fitted regression models in women were:

Mucogingival line:

$${L}^{*}=42.135-0.015\cdot {\textit{Age}}+7.902\cdot {\textit{Frequency of toothbrushing}} \left({\textit{per day}}\right)-1.710{\cdot {\textit{Frequency of toothbrushing}}}^{2}-1.176\cdot {\textit{Smoking habits}}+0.769\cdot {\textit{Long term medication}}$$

(range of prediction errors from − 21.5 to 14.3; residual standard deviation 5.7)

$${a}^{*}=31.840-0.025\cdot {\textit{Age}}-6.349\cdot {\textit{Frequency of toothbrushing}} \left({\textit{per day}}\right)+1.253{\cdot {\textit{Frequency of toothbrushing}}}^{2}-0.298\cdot {\textit{Smoking habits}}-0.380\cdot {\textit{Long term medication}}$$

(range of prediction errors from − 10.5 to 11.9; residual standard deviation 4.1)

$${b}^{*}=13.242-0.023\cdot {\textit{Age}}+3.435\cdot {\textit{Frequency of toothbrushing}} \left({\textit{per day}}\right)-0.868{\cdot {\textit{Frequency of toothbrushing}}}^{2}+0.495\cdot {\textit{Smoking habits}}+0.097\cdot {\textit{Long term medication}}$$

(range of prediction errors from − 7.4 to 6.6; residual standard deviation 2.7).

Middle zone:

$${L}^{*}=41.926+0.003\cdot {\textit{Age}}+8.691\cdot {\textit{Frequency of toothbrushing}} \left({\textit{per day}}\right)-1.873{\cdot {\textit{Frequency of toothbrushing}}}^{2}-1.159\cdot {\textit{Smoking habits}}+0.804\cdot {\textit{Long term medication}}$$

(range of prediction errors from − 19.7 to 12.3; residual standard deviation 5.3)

$${b}^{*}=14.017-0.029\cdot {\textit{Age}}+3.349\cdot {\textit{Frequency of toothbrushing}} \left({\textit{per day}}\right)-0.811{\cdot {\textit{Frequency of toothbrushing}}}^{2}+0.080\cdot {\textit{Smoking habits}}+0.328\cdot {\textit{Long term medication}}$$

(range of prediction errors from − 6.3 to 9.4; residual standard deviation 3.0).

Free gingival margin:

$${L}^{*}=44.153-0.009\cdot {\textit{Age}}+7.058\cdot {\textit{Frequency of toothbrushing}} \left({\textit{per day}}\right)-1.473{\cdot {\textit{Frequency of toothbrushing}}}^{2}-2.210\cdot {\textit{Smoking habits}}+0.312\cdot {\textit{Long term medication}}$$

(range of prediction errors from − 21.7 to 11.6; residual standard deviation 5.3).

In women, age and the behavioural habits analysed explain 7.6% of the variation in the L* coordinate at the mucogingival line, 9.6% in the middle zone, and 9.9% at the free gingival margin. The explained variation of the a* coordinate in the upper zone was 8.1%, while the explained variation of the b* coordinate in the upper and middle zones was 7.7% and 6.4%, respectively [44]. In women, toothbrushing had an inverse effect on the L* coordinate: lightness decreased as the frequency of toothbrushing increased in all three gingival zones, unlike the results in men. In contrast, the L* coordinate increased in women who were taking long-term medication, whereas taking long-term medication darkened the attached gingiva in men, in all three zones examined. At the mucogingival line, an increased frequency of toothbrushing resulted in an increased amount of red (a* coordinate) and a decreased amount of yellow (b* coordinate).

## Discussion

Prior studies [10, 24] found differences in gingival colour between men and women, and the present results support that finding, based solely on the significant changes in the b* colour coordinate (a bluer colour having been observed in men). We therefore reject the first null hypothesis.

In this research, analysis of the effect of the predictors (age, frequency of toothbrushing, smoking habits and long-term medication) on each colour coordinate was performed separately for each sex and gingival location, because it was posited that the effects on the distinct categories of these two variables might differ. This supposition was confirmed by the present results.

For both men and women, age and the behavioural habits examined had a significant—albeit limited—effect on gingival colour in the three zones, so we also reject the second null hypothesis of the study. Nevertheless, the effect differed in each sex. In men, age and the behavioural factors predicted 12.8% of the lightness at the mucogingival line, 10.2% in the middle zone and 14.9% at the free gingival margin. Meanwhile, in women, the frequency of toothbrushing was the main variable predicting lightness in all three gingival zones, albeit with less predictive power (mucogingival line—7.6%; middle zone—9.6%, and FGM—9.9%). In the female population, the a* (8.1%) and b* (7.7%) colour coordinates could also be weakly predicted at the mucogingival line, as could the b* (6.4%) coordinate in the middle zone. Neither the importance shown by each independent predictor variable nor the direction was homogeneous in men or women (frequency of toothbrushing, smoking habits and long-term medication). This makes it difficult to establish a behavioural pattern associated with gingival colour changes in healthy attached gingiva, also considering that the predictor variables provide information that is statistically significant. When assessing the aforementioned effects, it is worth bearing in mind that only a small number of study participants brushed their teeth 0 or 4 times per day. This means that the uncertainties when assessing the effects of increasing frequency of toothbrushing from 0 to 1 and from 2 to 4 times per day are very large, and the confidence intervals are therefore wider. Information on the type of long-term medication, how long the subject had been in treatment, the dosage and similar data would be valuable in order to fit the predictive model, but the range of possible responses is extremely wide. We therefore used the dichotomous variable of whether the participant was taking long-term medication or not, so as to explore its general effect. Most of the studies cited on the effects of taking medications refer to local melanin pigmentations [35, 37] and not to potential overall changes in colour coordinates. A comparison between the percentages of variation explained by regression models fitted to predict the colour of the teeth according to sex and age (45% of the total variation in the L* coordinate, 21% of the variation in the a* coordinate, and 17% of the variation in the b* coordinate) [41] and the percentages of variation explained by the present study’s models suggests that the former models predict gingival colour slightly less effectively. Nevertheless, the quality of the fitted models for the three gingival zones in this study cannot be directly compared to the predictive models used in other research, since no similar studies exist.

There is consensus on the fact that there are several anatomically distinct regions of gingival tissue [46, 47], which results in a non-uniform colour across these areas. The gradual reduction in gingival thickness from the attached gingiva to the free gingival margin leads to a progressive chromatic change that should be taken into account in order to satisfactorily reproduce the colour and texture of the gingiva. This chromatic difference between distinct areas of attached gingiva has been considered by most authors [10, 27]. Heydecke et al. [11] also recorded the colour of the interincisal papilla, whereas other publications have only noted the colour of the central part of the attached gingiva [17, 24, 48]. The statistically significant differences found between the sexes [10, 17, 27] have led to results being presented separately for men and women, although it should be noted that not all results point to these chromatic differences [11]. There are statistically significant differences in colour between men and women in the three zones of attached gingiva, and in all cases the b* coordinate is responsible for the differences. Despite this statistical significance, it should be stressed that there are no resulting clinical implications, since the colour difference in the b* coordinate (Δb*) is around 1.0 unit. This lack of clinical significance is because the difference falls below the perceptibility thresholds for the gingival colour space (3.1 units for ΔEab* and 2.1 units for ΔE00) [48]. Nevertheless, the fact that differences are detectable through spectrophotometry opens up a field of diagnostic applications for colour with respect to gingival health, which have yet to be explored.

The differentiated effect of the independent variables examined in men and women may be key in helping practitioners take into account the degree of complexity involved in reproducing the gingival colour map. It is worth noting that increasing the frequency of toothbrushing is associated with an increase in gingival lightness in men, while it has the opposite effect in women. Various hypotheses have been proposed to explain this, including the fact that women often brush their teeth more regularly and have distinct hormonal parameters to men. Additionally, women have a thinner gingival biotype [49], making the colour of the dental root more influential, while the thicker gingival biotype in men may respond differently to less frequent toothbrushing, which may prevent the colour of the underlying root from showing through. Another differential finding in men and women is that taking long-term medication results in the gingiva becoming darker in male subjects, while it becomes lighter in female patients. This may be informed by the fact that the long-term medication prescribed to women is usually different to that prescribed to men. Generally, male participants present with long-term medication related to hypertension, hypercholesterolemia and benign prostatic hyperplasia, while female participants more frequently take medication for depression, arthritis and the menopause. Despite the distinct results in men and women, what is clear is that taking medication has a significant impact on gingival colour. Given the extensive range of possible medications, it was decided to make this a dichotomous variable in the present study, which results in a loss of specificity. Future research is needed to explore which medications are most influential in the colour change, because dichotomising between individuals who take or do not take medication essentially divides the sample into older participants (who are more likely to take one or multiple medications) and younger individuals (who are not usually on long-term medication), and this distinction may be influential in itself.

In most scientific fields, predictive models are being developed, validated, updated and put into use, in order to help practitioners with their clinical decision-making. The predictive analysis techniques used to create models are primarily based on regression techniques. In light of the maximum R^2^ obtained (0.149 in men and 0.099 in women), it may be advisable to increase the depth and scope of the process of selecting independent variables, to increase the fit of the regression models. This type of prediction has the potential to provide colour coordinates based on the chromatic appearance of the natural gingiva: a key step in the process of improving the accuracy of gingival colour selection. This achievement would lead to greater patient satisfaction with the aesthetic results of gingival restorations, given that the colour difference between prosthetic restorations and natural gingival colour could be reduced to the extent that it falls below the clinical perceptibility threshold [48].

Most research agrees that subjective methods for colour selection have low repeatability, [50, 51] given that factors such as age, experience, ocular fatigue, and the type of illumination and colour guide material can have a negative influence on perception of colour. Spectrophotometers have come into use to eliminate subjectivity from the process of dental colour selection. They provide an objective method of recording colour coordinates, with an approximate reliability of 96% for dental tissue [52, 53], and almost perfect repeatability and reproducibility (ICC > 0.9) for gingival tissue [55]. Their use with gingival tissue has therefore become increasingly frequent [10, 38, 55], eliminating the errors involved in traditional visual comparisons. One of the advantages of spectrophotometry—the method used in this study—is that the instruments have a longer service life than colorimeters and are not affected by metamerism [56]. The spectrophotometer used (Spectroshade Micro MHT) has a large window that captures the whole of the object to be measured, making it less susceptible to the phenomenon of edge loss [54]. Spectrophotometers are therefore currently considered the most accurate, useful and flexible instruments available for determining colour in dentistry [52, 57]. The high levels of accuracy and objectivity provided by spectophotometry when quantifying gingival colour with colour coordinates may be extremely helpful for diagnosing periodontal or buccal diseases. Establishing a range of colour coordinates for healthy attached gingiva in each sex, race and age group would enable clinicians to identify potential inflammation, lesions or buccal pathology when colour coordinates fall outside these limits. This information would be of diagnostic use, with the additional advantage of being non-invasive, as colour measurement with spectrophotometry lacks adverse effects.

There is a lack of data on patient satisfaction with the gingival colour of fixed or removable prosthetic restorations but we can say that, while most studies point to a high level of satisfaction in patients who have received implant-supported restorations [58, 59], they usually have a significantly worse opinion of the final result in the soft tissue [60, 61]. Nevertheless, completely recreating the contours of the hard and soft tissue in a predictable manner when three-dimensional defects are present remains a challenge, despite the latest advances in regenerative periodontal and peri-implant surgery. Well-designed prosthetic gingival restorations can overcome the limitations of grafts, making them an option that should be considered at the start of the treatment process [62].

This cross-sectional study is exploratory, analysing the relationship between variables, and has not been designed to examine changes over time, for which purpose longitudinal studies would be needed. Nor does this research enable us to establish cause-effect relationships, which would require an experimental study. These results illustrate the need to examine the relevance of the variables analysed in greater depth, to design longitudinal studies [22], and to incorporate new variables (to determine gingival thickness/gingival biotype in millimetres [62], quantify the amount of medication, explore hormonal factors, etc.). Chromatic analysis of the interdental papilla would also contribute significantly to achieving the level of chromatic similarity required for fixed implant- and tooth-supported prostheses, so future studies should take this into consideration. It would also be interesting to assess whether explanatory capacity varies with the race of the patient, which would necessitate broadening the samples not only in terms of numbers but the racial variability of the population. The results obtained confirmed that the regression models produced do not provide very robust predictions of gingival colour changes (dental prediction models using a similar methodology are more reliable) and showed that age is not such an important factor as it is for dental colour changes.

It is now vital to confirm the practical and clinical utility of the proposed colour prediction models and to conduct similar studies on larger samples of individuals that include racial variability, so as to predict the optical behaviour of the gingiva precisely and enable practitioners to create gingival restorations that are as well-integrated as possible from a functional and aesthetic perspective. There is still a significant lack of knowledge on the natural evolution of the form, thickness, texture and colour of the gingiva that surrounds the natural teeth, and the factors that influence this. When considering a prosthetic gingival restoration as a treatment option, studying the chromatic optical properties of the gingiva is a key step in enabling practitioners to reproduce the gingiva prosthetically using the materials available: primarily acrylic or composite resins, and ceramics.

## Conclusions

1. the colours of gingiva in men contain a larger amount of blue, given that the values recorded for men were significantly lower than those recorded for women in the b* coordinate, but not in the other coordinates. This gingival colour difference has no clinical implications since it is not visible, falling below the gingival perceptibility thresholds.
2. In both sexes, age, smoking habits, frequency of toothbrushing and long-term medication have a modest capacity to explain gingival colour in all three zones of attached gingiva. The L* coordinate is the dependent variable that shows the greatest predictability. Much remains to be learned about the relative influence of each of the factors responsible for the significant variability in gingival colour.

## Data Availability

The data will be available upon motivated request.
